# Supplementary figures and images for: Maize-bean intercropping mediates reduction in arthropod intraguild predation better than low-intensity farming—Stable isotope evidence
Source: PLoS One. 2025 Aug 19;20(8):e0329756. doi: 10.1371/journal.pone.0329756 (PMC12364319; doi:10.1371/journal.pone.0329756)

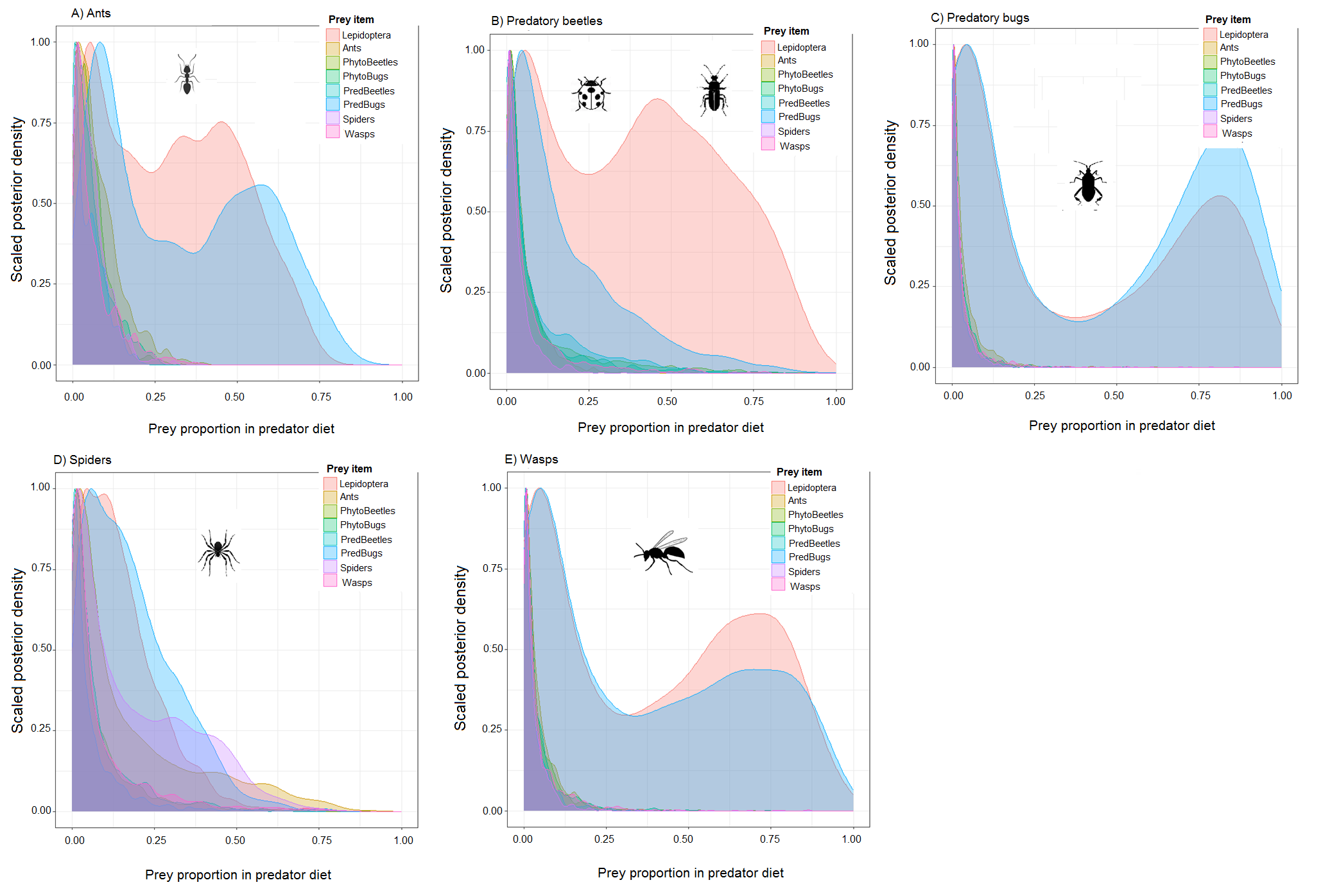

Supplement: S1 Fig — (TIF) [file pone.0329756.s001.tif]
