# Supplementary material for: Maize-bean intercropping mediates reduction in arthropod intraguild predation better than low-intensity farming—Stable isotope evidence
Source: PLoS One. 2025 Aug 19;20(8):e0329756. doi: 10.1371/journal.pone.0329756 (PMC12364319; doi:10.1371/journal.pone.0329756)
Supplement: S1 File — (PDF) [file pone.0329756.s002.pdf]

S1 File: Checklist of herbivorous and predatory arthropod representative families from which samples were drawn for stable isotopic analyses, including their distribution across the farming systems and cropping method

Farming system	Cropping method	Order	Family	Guild
Conventional	Intercropping	Araneae	Arachnidae	
Conventional	Monoculture	Araneae	Arachnidae	Predator
Conventional	Intercropping	Hymenoptera	Braconidae	Predator
Conventional	Intercropping	Coleoptera	Carabidae	Predator
Conventional	Monoculture	Coleoptera	Carabidae	Predator
Conventional	Intercropping	Hymenoptera	Chalcididae	Predator
Conventional	Monoculture	Coleoptera	Chrysomelidae	Predator
Conventional	Intercropping	Coleoptera	Coccinellidae	Predator
Conventional	Monoculture	Coleoptera	Coccinellidae	Predator
Conventional	Intercropping	Hymenoptera	Eulophidae	Predator
Conventional	Intercropping	Dermaptera	Forficulidae	Predator
Conventional	Monoculture	Dermaptera	Forficulidae	Predator
Conventional	Intercropping	Hymenoptera	Formicidae	Predator
Conventional	Monoculture	Hymenoptera	Formicidae	Predator
Conventional	Monoculture	Hymenoptera	Ichneumonidae	Predator
Conventional	Monoculture	Hymenoptera	Ichneumonidae	Predator
Conventional	Monoculture	Coleoptera	Lagriidae	Predator
Conventional	Monoculture	Coleoptera	Melyridae	Predator
Conventional	Intercropping	Coleoptera	Merylidae	Predator
Conventional	Monoculture	Coleoptera	Pentatomidae	Predator
Conventional	Monoculture	Hemiptera	Pentatomidae	Predator
Conventional	Monoculture	Hemiptera	Pentatomidae	Predator
Conventional	Intercropping	Hymenoptera	Sapygidae	Predator
Conventional	Intercropping	Hymenoptera	Scelionidae	Predator
Conventional	Monoculture	Hymenoptera	Scelionidae	Predator
Conventional	Intercropping	Hymenoptera	Scoliidae	Predator
Conventional	Intercropping	Dermaptera	Spongiphoridae	Predator
Conventional	Monoculture	Dermaptera	Spongiphoridae	Predator
Conventional	Intercropping	Dermaptera	Tenebrionidae	Predator
Organic	Intercropping	Araneae	Arachnidae	Predator
Organic	Monoculture	Araneae	Arachnidae	Predator
Organic	Intercropping	Hymenoptera	Braconidae	Predator
Organic	Intercropping	Coleoptera	Cantharidae	Predator
Organic	Intercropping	Coleoptera	Carabidae	Predator
Organic	Monoculture	Coleoptera	Carabidae	Predator
Organic	Monoculture	Coleoptera	Carabidae	Predator
Organic	Intercropping	Hymenoptera	Chalcididae	Predator
Organic	Monoculture	Coleoptera	Chrysomelidae	Predator
Organic	Intercropping	Coleoptera	Coccinellidae	Predator
Organic	Monoculture	Coleoptera	Coccinellidae	Predator
Organic	Intercropping	Dermaptera	Forficulidae	Predator
Organic	Monoculture	Dermaptera	Forficulidae	Predator
Organic	Intercropping	Hymenoptera	Formicidae	Predator

Organic	Monoculture	Hymenoptera	Formicidae	Predator
Organic	Intercropping	Hymenoptera	Ichneumonidae	Predator
Organic	Intercropping	Coleoptera	Lagriidae	Predator
Organic	Intercropping	Coleoptera	Melyridae	Predator
Organic	Monoculture	Hymenoptera	Mymaridae	Predator
Organic	Intercropping	Hemiptera	Pentatomidae	Predator
Organic	Monoculture	Hemiptera	Pentatomidae	Predator
Organic	Intercropping	Hymenoptera	Scelionidae	Predator
Organic	Monoculture	Hymenoptera	Scelionidae	Predator
Organic	Intercropping	Dermaptera	Spongiphoridae	Predator
Organic	Monoculture	Dermaptera	Spongiphoridae	Predator
