# Supplementary material for: Maize-bean intercropping mediates reduction in arthropod intraguild predation better than low-intensity farming—Stable isotope evidence
Source: PLoS One. 2025 Aug 19;20(8):e0329756. doi: 10.1371/journal.pone.0329756 (PMC12364319; doi:10.1371/journal.pone.0329756)
Supplement: S2 File — (PDF) [file pone.0329756.s003.pdf]

S2 Table. Topology of the selected study farms showing details of attributes based on agronomic practice criteria: (arming system and cropping method)

Agronomic practice		Farming system	
		Low intensity	Conventional
Cropping method	Monoculture	F1	F9
		F2	F10
		F3	F11
			F12
	Intercropped	F4	
		F5	F13
		F6	F14
		F7	F15
		F8	
