# Supplementary material for: Maize-bean intercropping mediates reduction in arthropod intraguild predation better than low-intensity farming—Stable isotope evidence
Source: PLoS One. 2025 Aug 19;20(8):e0329756. doi: 10.1371/journal.pone.0329756 (PMC12364319; doi:10.1371/journal.pone.0329756)
Supplement: Attachment — Submitted filename: PONE-D-25-26876 review report.docx [file pone.0329756.s004.docx]

**Review Report (Manuscript Number PONE-D-25-26876)**

**Title: Mitigating arthropod intraguild predation to enhance pest biocontrol better realized under maize-bean intercropping than through low-intensity farming**

**Overall report:**

This manuscript tackles a highly relevant and ecologically significant question: comparing the effectiveness of maize-bean intercropping versus low-intensity farming in mitigating arthropod intraguild predation (IGP) to improve pest biocontrol. The core research is sound, methodologically robust (particularly the use of stable isotope analysis), and addresses an important gap in sustainable agriculture for smallholder systems. While the findings are valuable and the study well-conceived, the presentation requires significant refinement to enhance clarity, focus, and impact.

**Comments:**

**Title:** The current title accurately reflects the study's focus but feels cumbersome. I suggest streamlining it for better readability and impact.

**Abstract:** The abstract provides a good overview but is quite dense and laden with technical jargon, making it challenging to parse quickly. Long sentences compound this issue. To strengthen it: **Improve Flow & Clarity:** Restructure for better separation between background, methods, key results, and conclusions. Use shorter sentences and simplify terminology where possible without losing scientific precision. **Highlight Key Quantification:** While some results are mentioned, emphasize the magnitude of significant findings more clearly. For instance, explicitly state the percentage increase in pest consumption under intercropping compared to the alternatives if possible, rather than just indicating "higher" rates. **Sharpen Implications:** Conclude with a clearer, more impactful statement on the practical significance of finding intercropping superior for reducing IGP and boosting biocontrol.

**Introduction:** The introduction sets a strong theoretical foundation, effectively covering pest biocontrol challenges, the concept of structural complexity, and the critical issue of IGP disrupting natural enemy efficacy. It compellingly argues for exploring alternatives like intercropping in smallholder maize systems. However, it tends towards excessive length and some repetition, which dilutes its punch. Crucially, while the objectives and hypotheses are present, they get somewhat lost in the detailed background. I strongly urge you to **condense the background narrative** and **state the specific research objectives and testable hypotheses clearly and succinctly at the end of the introduction** to provide a sharp focus for the reader.

**Materials and Methods:** This section demonstrates commendable methodological rigor, particularly the detailed field sampling and the sophisticated application of stable isotope analysis combined with Bayesian mixing models (MixSIAR) to unravel trophic interactions and IGP. However, readability is hampered in places: **Structure & Accessibility:** Some technical descriptions, especially around the statistical modeling, could be made more accessible. Consider adding clearer subheadings and potentially a schematic diagram outlining the overall experimental design and analytical workflow – this would be immensely helpful. **Taxon Selection Rationale:** Explicitly state why the specific arthropod taxa chosen for study were selected, linking this decision directly to the research questions on pest biocontrol and IGP dynamics in this system.

**Results and Discussion:** The findings presented are rich and complex, effectively using figures and tables to show taxon-specific variations in pest consumption and IGP across the different cropping and farming systems. The interpretation logically explains key patterns, such as enhanced pest consumption and reduced IGP in intercropped plots compared to monocultures under both farming intensities. The main structural issue is the **merging of Results and Discussion.** This creates lengthy passages intertwining data presentation with interpretation and speculation, which hinders clarity. **I strongly recommend separating these sections.** Present the core results objectively first. Then, in a distinct Discussion, interpret these results in the context of existing literature. While the ecological explanations are generally well-reasoned and referenced, some interpretations (e.g., specific foraging behaviors or indirect mechanisms) lean towards speculation without direct support from your data. Ensure the Discussion clearly distinguishes between conclusions strongly supported by your findings and hypotheses proposed for future testing.

**Conclusion:** The conclusion adequately summarizes the main finding intercropping's superiority over low-intensity farming alone in reducing IGP and boosting biocontrol via enhanced habitat complexity. However, it currently reads as somewhat repetitive of the abstract and lacks forward momentum. To strengthen it: **Focus on Key Takeaways:** Briefly reiterate the most significant outcome. **Emphasize Practical Value:** Explicitly state the applied implications. What does this mean for farmers, agronomists, or policymakers promoting sustainable practices? Recommend integrating maize-bean intercropping as a key strategy. **Address Minor Polish:** Proofread carefully for grammatical glitches and typos to ensure a professional finish.

**Recommendation:**

This study makes a substantial contribution to understanding how habitat management (specifically intercropping) can optimize natural pest control by modulating complex predator-predator interactions. The methodology, especially the trophic analysis, is a highlight. To elevate the manuscript for publication: **Sharpen the Title and Abstract** for clarity and impact. **Improve Methods readability** (structure, diagrams, rationale). **Separate Results and Discussion** sections. **Refine the Discussion** to clearly separate supported conclusions from speculation. **Perform thorough language editing** throughout.

Addressing these points will significantly enhance the manuscript's clarity, flow, and scientific impact, making its important findings more accessible and actionable.
