# Supplementary material for: Maize-bean intercropping mediates reduction in arthropod intraguild predation better than low-intensity farming—Stable isotope evidence
Source: PLoS One. 2025 Aug 19;20(8):e0329756. doi: 10.1371/journal.pone.0329756 (PMC12364319; doi:10.1371/journal.pone.0329756)
Supplement: Attachment — Submitted filename: How Editorial and Review concerns have been addressed.docx [file pone.0329756.s006.docx]

**How Editorial and Review comments, concerns and suggestions have been addressed**

**Editor Comments:**

Thank you for submitting your manuscript entitled "Mitigating arthropod intraguild predation to enhance pest biocontrol better realized under maize-bean intercropping than through low-intensity farming." After careful evaluation of the peer review reports, it is clear that your study addresses a relevant topic in sustainable agriculture and pest management. However, both reviewers have identified several important issues that need to be addressed before the manuscript can be considered for publication. These include concerns related to the clarity and detail of the methodology, the statistical analysis, the interpretation of results, and the overall presentation and flow of the discussion. I encourage you to revise the manuscript thoroughly in line with the reviewers' comments, ensuring that all points are clearly addressed and justified. Please provide a detailed point-by-point response letter with your revised submission to facilitate re-evaluation. We look forward to receiving your revised manuscript

***AUTHORS’ RESPONSE:*** We hank he Editor for comments and the chance for us to revise and re-submit our article for re-evaluation. We have now revised te manuscript thoroughly by taking into account all the comments and concerns, and incorporating the suggested changes from the reviewers, presenting our responses in a point-by-point form according to the details appended further below and submitted along with our revised paper.

In summary, we have made the following major changes:

1. The title has now been modified to make it more concise and explicit about the study’s objectives and focus
2. The abstract has also been re-written with a clearer highlights of the key findings, especially the quantitative aspects of the results
3. The Results and Discussion sections have been separated so as to distinguish each of the findings (Results) from their respective interpretations further down, putting these within the context of other studies (Discussion)
4. We have re-written Discussion to make our recommendations less prescriptive
5. The hypotheses have now been more clearly/specifically and testably re-stated as follows:
6. We have now provided greater clarity on sampling protocol and balance in sample farm selectivity and design, as well as provided a new table to illustrate this
7. In analyses, we have now clarified the Bayesian statistical modeling tool MixSIAR, provided comparisons of observed patterns in isotopic test results for consumer-to-food source relations across the various attributes (predators and pests or intraguild prey) and also across levels of the various treatment factors (farming system and cropping method). In addition to and to more clearly and defensibly support these statistical results, we have now also included test results showing corresponding posterior probability plots, food proportion medians and 95% credible intervals (from lower limits of 2.5% to upper limits of 97.5%). These rae provided both in text as well as in tables and density plots, and now more clearly illustrate differences reported in Results and elaborated in Discussion.
8. We have now acknowledged that in addition to direct feeding interactions between predatory arthropod taxa and their pest and intraguild prey as revealed by isotopic test results, it is possible that some of the diet carbon may have been obtained through scavenging on carcasses of such prey, and also potentially from detritivorous feeding on decomposing tissue residues in the soil
9. We have now checked the articles and corrected for grammatical and typographic errors
10. We have now ensured that all references cited are listed in bibliography and vice versa

A more detailed account of our responses to review comments are presented below, with our responses highlighted in green

[Note: HTML markup is below. Please do not edit.]

REVIEWERS' COMMENTS GENERAL:

Reviewer's Responses to Questions

**Comments to the Author**

1. Is the manuscript technically sound, and do the data support the conclusions?

The manuscript must describe a technically sound piece of scientific research with data that supports the conclusions. Experiments must have been conducted rigorously, with appropriate controls, replication, and sample sizes. The conclusions must be drawn appropriately based on the data presented.

Reviewer #1: Yes

Reviewer #2: Yes

2. Has the statistical analysis been performed appropriately and rigorously?

Reviewer #1: Yes

Reviewer #2: No

3. Have the authors made all data underlying the findings in their manuscript fully available?

The [PLOS Data policy](http://track.editorialmanager.com/CL0/http:%2F%2Fwww.plosone.org%2Fstatic%2Fpolicies.action%23sharing/1/010f0197829cd070-b3868c0f-e321-4865-86cb-c34f34862001-000000/KIvRzhL3mpzhBdcSYi0PbRIOtPUWimpXj4Jhyapgqtc=216) requires authors to make all data underlying the findings described in their manuscript fully available without restriction, with rare exception (please refer to the Data Availability Statement in the manuscript PDF file). The data should be provided as part of the manuscript or its supporting information, or deposited to a public repository. For example, in addition to summary statistics, the data points behind means, medians and variance measures should be available. If there are restrictions on publicly sharing data—e.g. participant privacy or use of data from a third party—those must be specified.

Reviewer #1: Yes

Reviewer #2: Yes

4. Is the manuscript presented in an intelligible fashion and written in standard English?

PLOS ONE does not copyedit accepted manuscripts, so the language in submitted articles must be clear, correct, and unambiguous. Any typographical or grammatical errors should be corrected at revision, so please note any specific errors here.

Reviewer #1: Yes

Reviewer #2: Yes

5. Review Comments to the Author

Please use the space provided to explain your answers to the questions above. You may also include additional comments for the author, including concerns about dual publication, research ethics, or publication ethics. (Please upload your review as an attachment if it exceeds 20,000 characters)

Reviewer #1:  Report

***REVIEWER COMMENT/SUGGESTIONS:*** This study makes a strong contribution to understanding how farming practices influence intraguild predation (IGP) and arthropod-mediated pest control in maize systems. The application of stable isotope analysis to quantify trophic interactions is particularly innovative and adds significant ecological depth. Your core finding that maize-bean intercropping surpasses low-intensity farming alone in reducing IGP and enhancing pest suppression offers novel and highly relevant insights for sustainable agriculture, especially within smallholder contexts. To maximize the manuscript's clarity and impact for readers, several areas need refinement.

***AUTHORS’ RESPONSE:*** We greatly appreciate this positive overall assessment by the Reviewer. The article has now been revised and edited according to the Refviewer’s recommendations, as detailed below

***REVIEWER COMMENT/SUGGESTIONS:*** The title and abstract require streamlining to enhance conciseness and explicitly highlight key quantitative outcomes.

***AUTHORS’ RESPONSE:***

11. The title has now been modified to make it more concise and explicit about the study’s objectives and focus
12. The abstract has also been re-written with a clearer highlights of the key findings, especially the quantitative aspects of the results

***REVIEWER COMMENT/SUGGESTIONS:*** While the introduction provides solid background, it feels overly dense; sharpening the focus and concluding with a clearer, succinct statement of objectives and hypotheses would greatly improve its direction.

***AUTHORS’ RESPONSE:*** We have now re-written the Introduction in a way that is much under-densed and instead more closely focused on the subject matter, surveying relevant existing literature and placing our work where it fills a knowledge gap, before ending with a more succinct statement of our clearer objectives and what we hypothesized

***REVIEWER COMMENT/SUGGESTIONS:*** Similarly, merging the results and discussion sections creates confusion; separating them would allow for clearer data presentation followed by distinct interpretation.

***AUTHORS’ RESPONSE:*** The Results and Discussion sections have been separated so as to distinguish each of the findings (Results) from their respective interpretations further down, putting these within the context of other studies (Discussion)

***REVIEWER COMMENT/SUGGESTIONS:*** Some ecological explanations, particularly those addressing underlying behavioral mechanisms, currently lean towards speculation and should be framed more cautiously as hypotheses for further testing.

***AUTHORS’ RESPONSE:*** The reviewer did not name those explanations in specific, however, we have now re-written those parts the Discussion in where some of our arguments might have earlier tended towards speculation, and reworded them to be less finalistic, definitive, deterministic or prescriptive, and we have instead advanced standpoints that provide room for inviting further investigations on such postulations and interpreted positions

***REVIEWER COMMENT/SUGGESTIONS:*** Finally, strengthening the conclusion to provide actionable guidance for farmers or policymakers on implementing intercropping would significantly enhance the study's practical relevance.

***AUTHORS’ RESPONSE:*** The Conclusion subsection has now been restructured to give it a more prescriptive approach for farm managers and policymakers

***REVIEWER COMMENT/SUGGESTIONS:*** Addressing these points will elevate an already valuable piece of research, ensuring its important findings on optimizing biocontrol through habitat management offer actionable insights for sustainable farming practices.

***AUTHORS’ RESPONSE:*** We thank the reviewer for this positive verdict on the potential value our paper and the findings in it, and we hope that by have now addressed their concerns and incorporated the suggested changes to a sufficient extent.

Reviewer #2: Reviewer Report
Manuscript Title:
Mitigating arthropod intraguild predation to enhance pest biocontrol better realized under maize-bean intercropping than through low-intensity farming
Manuscript Number:
PONE-D-25-26876
***REVIEWER COMMENT/SUGGESTIONS:*** Recommendation: Major Revision

***AUTHORS’ RESPONSE:*** We greatly appreciate this positive overall assessment by the Reviewer. The article has now been revised and edited according to the Refviewer’s recommendations, as detailed below
________________________________________
***REVIEWER COMMENT/SUGGESTIONS:***

General Assessment
This manuscript presents an important contribution to the field of agroecology and biological pest control by evaluating the role of habitat complexity (intercropping and low-intensity farming) in mitigating intraguild predation (IGP) among arthropod natural enemies using stable isotope analysis (SIA). The topic is timely and relevant, particularly for smallholder systems in the Global South.
The experimental approach using δ¹³C and δ¹⁵N isotopic signatures is well-justified, and the general structure of the manuscript is clear. However, significant improvements are required in hypothesis framing, description of methods, and interpretation of results to enhance scientific rigor and clarity.
________________________________________
Major Comments
***REVIEWER COMMENT/SUGGESTIONS:*** 1. Hypothesis Framing Needs Clarification
o The hypotheses presented are not clearly formulated or testable. The phrasing is long and ambiguous (lines 115–120).
o Recommendation: Clearly state the hypotheses at the end of the Introduction, using specific, testable language. For example:
“We hypothesized that (1) intercropping and low-intensity farming increase pest consumption and reduce intraguild predation, and (2) intraguild predation among predator taxa is not taxon-specific.”

***AUTHORS’ RESPONSE:*** The hypotheses have now been more specifically and testably re-stated as follows: “*We hypothesized that 1) Intercropping and low-intensity farming increase pest consumption and reduce intraguild predation pressure; 2) Prey selectivity amongst predator taxa is not taxon-specific”.*

***REVIEWER COMMENT/SUGGESTIONS:*** 2. Sampling Design Unclear and Unbalanced
o It is not clearly stated how many replicates exist per treatment combination (monoculture/intercrop × conventional/low-intensity), and the number appears unbalanced.
o Recommendation: Add a table summarizing the number of farms per treatment. Explain how unbalanced design, if present, was addressed analytically.

***AUTHORS’ RESPONSE:*** We have stated more clearly that the study farms were a total of 18, and of these, 3 were monoculture organic farms, 5 were intercropped organic, 4 were monoculture conventional and 3 were intercropped conventional. Therefore, although through this combination, the 7 conventional versus 8 organic were counterbalanced by the 8 intercropped vs 7 monoculture. Our analyses were based on comparing organic to conventional (total 15) and intercropped to monocropped ((total 15), **rather than the interactive pairwise interplays** between these treatments, which is the only case in which balancing might become a statistical concern. In addition, any potential balance-related statistical biases were further minimized by the fact that sampling was conducted at several times (multiple crop stages and multiple cropping seasons), which were then ultimately averaged/pooled rather than analyzed separately. We have now also added a supplementary/supporting information table (S2_File) outlining the details of study farm biotopes

***REVIEWER COMMENT/SUGGESTIONS:*** 3. Statistical Comparisons Are Lacking
o While isotopic results are presented, there is no mention of credible interval overlap or formal tests of differences between treatments or taxa.
o Recommendation: Use posterior probability comparisons, credible interval overlap, or other Bayesian inference tools to validate statements of statistical difference.

***AUTHORS’ RESPONSE:*** To add to the credible intervals for results that we already presented from MixSIAR Bayesian modeling in Table 1 for most significant intraguild predation patterns (diet contribution levels), we have now also added two new tables (Table 2 and Table 3) for estimated patterns of overall contributions of each prey (pest or predator) to diets of their consumers, together with respective values for medians and 95% credible intervals (lower as well as upper limits). We have also added a supplementary figure (S1 Fig) to the supporting material, showing the MixSIAR model posterior density plots, complete with the various patterns of 95% credible interval overlaps in food source contributions to the diets of their respective consumers Table 3, in particular, indcates the effects of the treatments (farming system and cropping method. Due to the large volume of test results usually generated from a typical MixSIAR Bayesian modeling (tens of tables and figures at a time), we are not able to present all statistical data in the results. Therefore we hope the Reviewer would concur with us that as a form of Bayesian modeling procedure, MixSIAR is a formal statistical analysis in itself for predicting probability distributions (in this case diverse food-source options contributing to diets of consumers) different from/alternative to the classical goodness-of-fit-to-mean approaches testing for p-values within specified confidence limits. Therefore for MixSIAR, posterior density overlap plots of the estimated median probabilities, accompanied by 95% credible intervals, which we have now added, should be sufficiently defensible.

***REVIEWER COMMENT/SUGGESTIONS:*** 4. Overinterpretation of SIA Results
o The manuscript often interprets stable isotope results as direct feeding relationships (e.g., scavenging vs. predation is not distinguished).
o Recommendation: Acknowledge this limitation in both Methods and Discussion. See Post (2002), Layman et al. (2012) for best practices.

***AUTHORS’ RESPONSE:*** We have now duly acknowledged in both Methods, and (right at the state of Discussion) sections, that scavenging might have added to the overall diet carbon intake by intraguild predators, but added that this might not have made substantial overall contributions. We have included relevant literature in support. Furthermore, among the species/groups of predators examined in the study and which are most likely to be occasionally associated with scavenging (spiders, ants, predatory bugs) or detritivory (ants, ground beetles), none of them is a obligate/predominantly scavengers or detritivores. Therefore contribution of these feeding streams was unlikely to have contributed overwhelmingly to overall feeding linkages. Furthermore the study did not entail quantitative analysis of direct predation and the other feeding pathways separately, and therefore interpretation of isotopic signatures is taken to account for all the pathway options
________________________________________
Minor Comments
***REVIEWER COMMENT/SUGGESTIONS:*** 1. Grammar and Typographical Issues
o Several typographical errors throughout the paper (e.g., "concentualization", “low-intensityally”) detract from clarity.

o Recommendation: Thorough proofreading is required.

***AUTHORS’ RESPONSE:*** These have now all been duly corrected

***REVIEWER COMMENT/SUGGESTIONS:*** 2. Figures and Tables

o Recommendation: Figure 1 quality needs to be improved to make it clear and readable.

***AUTHORS’ RESPONSE:*** Figure 1 quality is now much improved and is clearer and more legible. We have also disentangled the former Figure 1 and expanded it into four instead of two panels so as to more clearly illustrate the results of pest consumption and intraguild predation separately.

***REVIEWER COMMENT/SUGGESTIONS:*** 3. Ethics and Limitations
o The “Limitations” section should better acknowledge potential external sources of error (e.g., unaccounted prey taxa, spatial spillover).
o Recommendation: Strengthen this section with a more balanced reflection on SIA scope and limitations.

***AUTHORS’ RESPONSE:*** The Study limitations section has now been re-written to provide a clearer picture of the potential issued that might not necessarily have been addressed fully by the study, as well as the potential limiationsnans cope of stable isotope analytical results

***REVIEWER COMMENT/SUGGESTIONS:*** 4. Reference Formatting
o Several references contain inconsistencies or broken URLs. Few references like Blitzer et al 2012 have been included in bibliography but not cited.
o Recommendation: Carefully format references according to PLOS ONE guidelines.

***AUTHORS’ RESPONSE:***

1. All authorities cited in-text are now listed in References and vice versa
2. All references are now formatted according to PLOS ONE guidelines
3. All previous broken urls have now been re-joined

6. PLOS authors have the option to publish the peer review history of their article ([what does this mean?](https://track.editorialmanager.com/CL0/https:%2F%2Fjournals.plos.org%2Fplosone%2Fs%2Feditorial-and-peer-review-process%23loc-peer-review-history/1/010f0197829cd070-b3868c0f-e321-4865-86cb-c34f34862001-000000/JzAwqWLNTkNDWEuJj92-90JBquIc7t9koJPSFyWyuP8=216)). If published, this will include your full peer review and any attached files.


If you choose “no”, your identity will remain anonymous but your review may still be made public.


**Do you want your identity to be public for this peer review?** For information about this choice, including consent withdrawal, please see our [Privacy Policy](https://track.editorialmanager.com/CL0/https:%2F%2Fwww.plos.org%2Fprivacy-policy/1/010f0197829cd070-b3868c0f-e321-4865-86cb-c34f34862001-000000/9Th1UeGboZBQXOg4NlBsOfsi36_WupkhkwjVNFab7gg=216).

Reviewer #1: **Yes:** Muhammad Imran

Reviewer #2: **Yes:** Farrukh Baig
